# *Pandanus amaryllifolius* Exhibits In Vitro Anti-Amyloidogenic Activity and Promotes Neuroprotective Effects in Amyloid-β-Induced SH-SY5Y Cells

**DOI:** 10.3390/nu14193962

**Published:** 2022-09-24

**Authors:** Mario A. Tan, Hayato Ishikawa, Seong Soo A. An

**Affiliations:** 1 College of Science and Research Center for the Natural and Applied Sciences, University of Santo Tomas, Manila 1015, Philippines; 2 Graduate School of Pharmaceutical Sciences, Chiba University, 1-8-1 Inohana, Chuo-ku, Chiba 260-8675, Japan; 3 Department of Bionano Technology, Bionano Research Institute, Gachon University, Seongnam-si 1342, Gyeonggi-do, Korea

**Keywords:** Alzheimer’s disease, amyloid-beta, neuroprotection, nicotinamide, *Pandanus amaryllifolius*, Thioflavin T, high-throughput screening multimer detection system (MDS-HTS) assay

## Abstract

Accumulation of amyloid-*beta* (Aβ) plaques leading to oxidative stress, mitochondrial damage, and cell death is one of the most accepted pathological hallmarks of Alzheimer’s disease (AD). *Pandanus amaryllifolius*, commonly recognized as fragrant screw pine due to its characteristic smell, is widely distributed in Southeast Asia and is consumed as a food flavor. In search for potential anti-AD agents from terrestrial sources, *P. amaryllifolius* was explored for its in vitro anti-amyloidogenic and neuroprotective effects. Thioflavin T (ThT) assay and the high-throughput screening multimer detection system (MDS-HTS) assay were used to evaluate the extracts’ potential to inhibit Aβ aggregations and oligomerizations, respectively. The crude alcoholic extract (CAE, 50 μg/mL) and crude base extract (CBE, 50 μg/mL) obstructed the Aβ aggregation. Interestingly, results revealed that only CBE inhibited the Aβ nucleation at 100 μg/mL. Both CAE and CBE also restored the cell viability, reduced the level of reactive oxygen species, and reversed the mitochondrial dysfunctions at 10 and 20 μg/mL extract concentrations in Aβ-insulted SY-SY5Y cells. In addition, the unprecedented isolation of nicotinamide from *P. amaryllifolius* CBE is a remarkable discovery as one of its potential bioactive constituents against AD. Hence, our results provided new insights into the promising potential of *P. amaryllifolius* extracts against AD and further exploration of other prospective bioactive constituents.

## 1. Introduction

Alzheimer’s disease (AD) is a progressive neurodegenerative disorder commonly depicted by memory loss, mental dysfunctions, cognitive and learning impairments, and emotional instability [1]. With global prevalence as the seventh cause of death in 2020 and 2021, it is estimated that 44 million people worldwide are affected by AD [2]. With a complex plethora of etiologies, the excessive formation and deposition of amyloid-*beta* (Aβ) plaques leading to oxidative stress, mitochondrial dysfunctions, and eventual nerve cell death and tissue loss are the most accepted pathological hallmark of AD [3]. Currently, US FDA approved the acetylcholinesterase inhibitors donepezil galantamine, tacrine, and rivastigmine; the *N*-methyl-D-aspartate (NMDA) receptor antagonist memantine; and aducanumab, a monoclonal antibody targeting the aggregation of Aβ [4,5], to combat AD. However, these drugs only provide symptomatic relief of AD and come with adverse side effects [5,6]. Moreover, treatment of AD utilizing aducanumab would be very expensive at an annual price of USD 56,000 [7]. With the rapid growth of people worldwide with AD, the discovery of potential drugs and medicinal plants with minimal adverse effects, cost-effective, and may help to alleviate the progression of AD is warranted. Plants have been regarded as primary sources of diverse and pharmacologically important metabolites used in drug discovery research for the treatment of various diseases, including AD [8,9,10,11].

*Pandanus amaryllifolius*, commonly known as fragrant screw pine, is one of the 700 species of the Genus *Pandanus* widely distributed in tropical and sub-tropical environments such as the Southeast Asian countries, India, Taiwan, Papua New Guinea, and Sri Lanka [12]. It is utilized in Philippine traditional folk medicine to treat stomachache, high blood pressure, urinary tract infection, and kidney illness [13]. It is also used in cooking to impart color, flavor, and a distinct smell identified as 2-acetyl-1-pyrroline [14,15]. Phytochemical investigations on the leaf extract elaborated the identification of diverse alkaloid structures [16] and flavonoid and phenolic compounds [17,18].

In our continuing search for Philippine medicinal plants and their constituents with inhibitory effects against Aβ toxicity in vitro [19,20,21,22,23], we herein describe the neuroprotective effects and inhibition of Aβ aggregations and oligomerizations of *P. amaryllifolius* crude alcoholic (CAE) and crude base (CBE) extracts. The unprecedented isolation of nicotinamide from *P. amaryllifolius*, as a potential bioactive constituent of the crude base extract, is also reported.

## 2. Materials and Methods

### 2.1. Plant Material

Fresh, matured *P. amaryllifolius* leaves were collected in Santa Maria, Bulacan, Philippines, in April 2017. Leaves were authenticated at the Botany Division, National Museum of the Philippines (Control #17-04-533). Voucher specimens were deposited at the UST Herbarium, Research Center for Natural and Applied Sciences (USTH-3728).

### 2.2. Extraction of the Crude Extracts

Air-dried, ground *P. amaryllifolius* leaves (2 kg) were extracted with distilled MeOH five times and filtered. The combined filtrates were concentrated under reduced pressure yielding the crude alcoholic extract (CAE, 178 g). A portion of the CAE (170 g) was dissolved in 1 M HCl and partitioned with EtOAc thrice. The aqueous layer was basified to pH 9 using Na_2_CO_3_. The basified aqueous layer was extracted with 5% MeOH in CHCl_3_ five times. The combined organic layer was dried with anhydrous Na_2_SO_4_ and concentrated in vacuo yielding the crude base extract (CBE, 3.3 g).

### 2.3. Thioflavin T (ThT) Assay

ThT assay was used to evaluate the inhibition of Aβ aggregation of the *P. amaryllifolius* extracts and phenol red (positive control) as previously described [21,22,23]. Briefly, Aβ42 (Aggresure™ AnaSpec, Fremont, CA, USA) in PBS was mixed with or without the *P. amaryllifolius* extracts or phenol red for 24 h at 37 °C. After the addition of ThT solution, the mixture was incubated for 15 min, and the fluorescence signal (Ex 450 nm; Em 510 nm) was measured. The percentage inhibition was calculated using the equation: [(1 − *I*_Fi_/*I*_Fc_) × 100%], where *I*_Fi_ (with inhibitor) and *I*_Fc_ (without inhibitor) are the fluorescence signals after subtracting the background signal of the ThT solution.

### 2.4. High-Throughput Screening of Multimer Detection System (MDS-HTS) Assay

The ability of *P. amaryllifolius* extracts to inhibit the Aβ oligomerization was evaluated by the MDS-HTS assay (PeopleBio Inc., Sungnamsi, Gyeonggido, Korea), as previously described [24]. Aβ_42_ (200 μg/mL), the crude extracts (100 μg/mL), and BPL-1 (100 μM, positive control) were dissolved in PBS and were utilized, following an enzyme-linked immunosorbent assay method [24,25,26]. Briefly, the 1000-times-diluted mixture of Aβ_42_ and the extracts in PBST (100 μL) were added to the antibody-coated wells of a 96-well microtiter plate and incubated for 1 h at RT. Detection antibody with conjugated horseradish peroxidase (HRP, 100 μL) was employed to spot the Aβ oligomer attached to the capture antibody. After washing with washing buffer, a solution of 3,3′,5,5′-tetramethylbenzidine (100 μL) was added and incubated at RT for 30 min. A stop solution (50 μL) was finally added, and the optical density was measured after 24 h. The significant difference (*p* < 0.05) against the negative control was determined.

### 2.5. Cell Culture

Neuroblastoma SH-SY5Y cells (ATCC CRL-2266) were obtained from the American Type Culture Collection (Manasas, VA, USA) and maintained in DMEM supplemented with 10% FBS and 1% kanamycin/penicillin at 37 °C and 5% CO_2_. Cells at 80–90% confluency were used in the experiments.

### 2.6. Cell Cytotoxicity and Neuroprotection Assay

SH-SY5Y cells (1 × 10^4^ cells/well) were plated in 96-well plate and acclimatized for 24 h. Then, the cells were treated with 50, 20, 10, and 1 μg/mL *P. amaryllifolius* extracts for 24 h. After treatment, cells were washed with PBS, followed by the addition of 100 μL fresh media and incubation for 30 min. The cell viability was assessed following the ATP Luminescence (CellTiter-Glo^®^) (Promega, Madison, WI, USA) method as previously described [22,23]. The cell viability was expressed as % of the control cells (untreated).

For the neuroprotective experiment, SH-SY5Y cells (1 × 10^4^ cells/well) were sub-cultured in 96-well plate and incubated for 24 h. After incubation, cells were then treated with the 20, 10, and 1 μg/mL *P. amaryllifolius* extracts for 6 h. This was followed by Aβ (10 μM) treatment for 24 h. The % cell viability was determined in triplicate experiments using the ATP Luminescence (CellTiter-Glo^®^) method as previously described [22,23].

### 2.7. Determination of Intracellular Reactive Oxygen Species (ROS)

After 24 h incubation, SH-SY5Y (1 × 10^4^ cells/well) cells were pre-treated with 20, 10, and 1 μg/mL *P. amaryllifolius* extracts for 6 h and continued with 10 μM Aβ for 24 h. After treatment, cells were then incubated with 25 μM H_2_DCFDA (Sigma Aldrich, St. Louis, MO, USA) for 2 h in 37 °C oven. Fluorescence intensity (Ex 495 nm, Em 520 nm) was measured, and the ROS level was calculated as a percentage of the control cells in triplicate experiments.

### 2.8. Mitochondrial Membrane Potential (∆Ψm) Assay

Following 24 h incubation, SH-SY5Y (1 × 10^4^ cells/well) cells were pre-treated with 20, 10, and 1 μg/mL *P. amaryllifolius* extracts for 6 h and incubated with 10 μM Aβ for 24 h. The process was followed by the addition of 1 μM TMRE staining solution (Abcam TMRE mitochondrial membrane kit) and incubation at 37 °C for 1 h. The fluorescence (Ex 549 nm, Em 575 nm) was measured, and the ∆Ψm was calculated as a percentage of the untreated control (untreated) cells in triplicate experiments.

### 2.9. Isolation of Nicotinamide (1)

**Figure 1:**
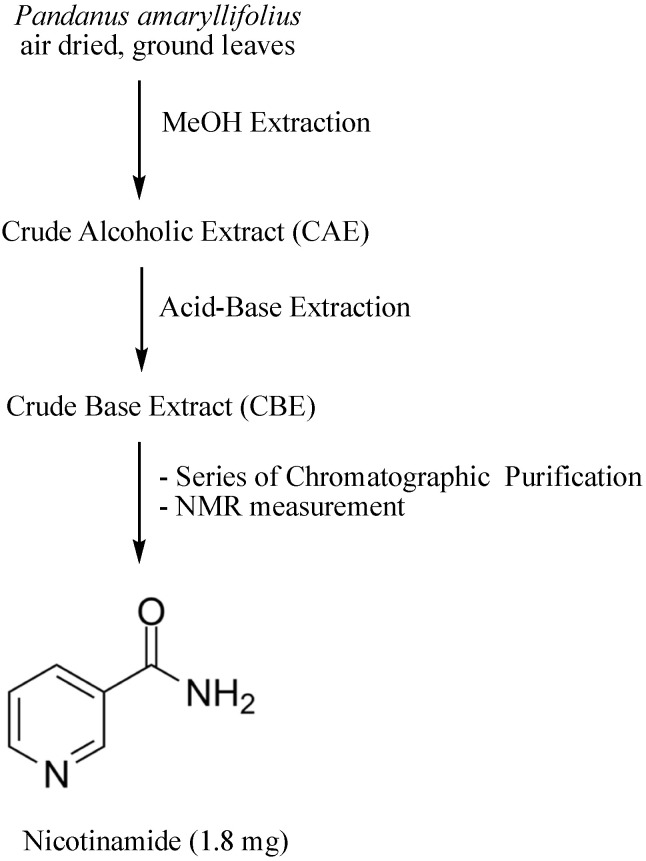
Schematic diagram on the isolation and identification of Nicotinamide.

CBE (3 g) was initially separated by silica gel flash column chromatography using CHCl_3_/MeOH mixtures in increasing polarity. The collected fractions were subjected to thin layer chromatography to obtain 4 pooled fractions, CBE1–CBE4. CBE 3 was partitioned by amino silica gel column chromatography using increasing polarity of EtOAc in hexane, neat EtOAC, 1:1 EtOAc/MeOH, and neat MeOH. The fraction eluting in neat EtOAc (10 mg) was subjected to MPLC using 1:1 CHCl_3_/acetone as eluent to obtain nicotinamide as white crystals (1.8 mg). The schematic diagram of the isolation and identification of nicotinamide from *P. amaryllifolius* is presented in Figure 1. The ^1^H NMR, ^13^C NMR, and LCMS spectra of nicotinamide are found in the Supplementary Material.

Nicotinamide: white crystals; ^1^H NMR (CDCl_3_, 400 MHz) δ 9.02 (1H, d, *J* = 2.0 Hz), 8.77 (1H, dd, *J* = 5.0, 1.8 Hz), 8.18 (1H, dt, *J* = 8.0, 2.0 Hz), 7.43 (1H, dd, *J* = 8.0, 5.0 Hz); ^13^C NMR (CDCl_3_, 150 MHz) δ 167.1 (C=O), 152.8 (CH), 148.2 (CH), 135.5 (CH), 129.6 (C), 123.6 (CH).

### 2.10. General Considerations

^1^H-NMR was recorded on an ECZ 400 FT-NMR spectrometer, and ^13^C-NMR spectra were recorded on an ECZ 600 FT-NMR spectrometer using CDCl_3_ as solvent and TMS as internal standard. Thin layer chromatography was performed using Merck 60 F254 precoated silica gel plates (0.25 mm thickness). UV_254_, followed by Dragendorff reagent, was used for visualization. Medium-pressure liquid chromatography (MPLC) was accomplished using a silica gel prepacked column CPS-HS-221-05. PerkinElmer Victor-3^®^ multi-plate reader was used to measure the luminescence, fluorescence, or optical density in the biological assays.

### 2.11. Statistical Analysis

The quantitative data were reported as mean ± SD of at least three experiments. Statistical analysis was determined by one-way ANOVA (GraphPad Prism 5 software package, version 5.02, GraphPad Software Inc., San Diego, CA, USA), with the statistical significance considered at *p* < 0.05.

## 3. Results

### 3.1. Inhibitory Effect on the Aggregation and Oligomerization of Amyloid-Beta

The anti-amyloidogenic potential of *P. amaryllifolius* CAE and CBE fractions were elucidated using the ThT and MDS-HTS assays (Table 1). ThT binds to the Aβ aggregates resulting in an increase in the fluorescence signal. With the presence of potential inhibitors, the formation of Aβ fibrils and aggregates is prevented, thus, resulting in a decrease in the fluorescence signal [27]. As shown in Table 1, both extracts inhibited the Aβ aggregation. However, CBE revealed a stronger inhibitory effect at 89.53% in comparison to the positive control (70.06%) and CAE (73.67%). These results were also manifested in the MDS-HTS assay for screening Aβ breakers, which was an ELISA-based technique utilizing a capturing antibody against Aβ and a detecting antibody (horseradish peroxidase antibody, HRP), which allowed the selective detection of Aβ oligomers [26,27]. The inhibitors would break up the Aβ aggregates and reduce the concentrations of the captured oligomers, thus, resulting in a reduction of signal and preventing Aβ oligomerization [24,25,26]. The alkaloidal extract (CBE) showed a significant reduction in Aβ oligomer formation against the negative control. On the contrary, a significant difference was also observed with the CAE against the negative control but may further promote oligomerization as indicated in the increase in optical density.

### 3.2. Cell Cytotoxicity and Neuroprotective effects of P. amaryllifolius Extracts

**Figure 2:**
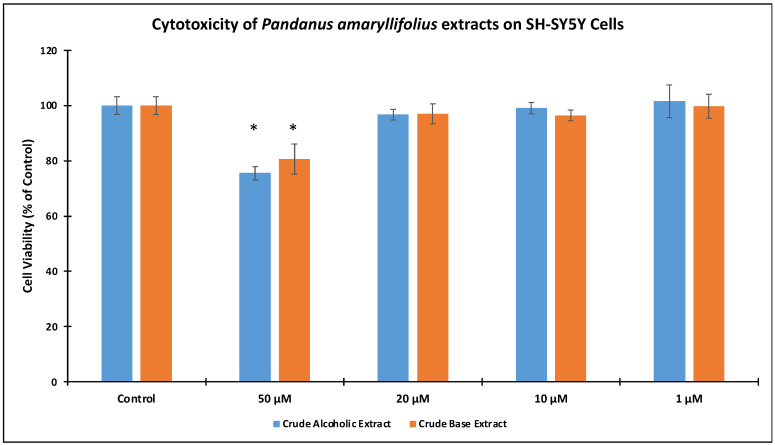
Cell cytotoxicity of *Pandanus amaryllifolius* Crude Alcoholic Extract (CAE) and Crude Base Extract (CBE). SH-SY5Y cells were treated with 1–50 μg/mL extract concentration and the cell viability were evaluated by ATP Luminescence assay. “Control” refers to untreated cells. The (*) showed significant difference with the control cells at *p* < 0.05.

Prior to the neuroprotective experiments, SH-SY5Y cell viability of the crude extracts at 1, 10, 20, and 50 μg/mL concentrations were monitored (Figure 2). Results indicated that >95% cell viability was exhibited when the cells were treated with 1–20 μg/mL extract concentrations. However, 75.56 ± 2.97% for CAE and 80.64 ± 1.78% for cell viability were observed at 50 μg/mL. These cell viabilities were significantly different from the control (untreated) cells at *p* < 0.05. Hence, succeeding experiments utilized 1, 10, and 20 μg/mL concentrations.

**Figure 3:**
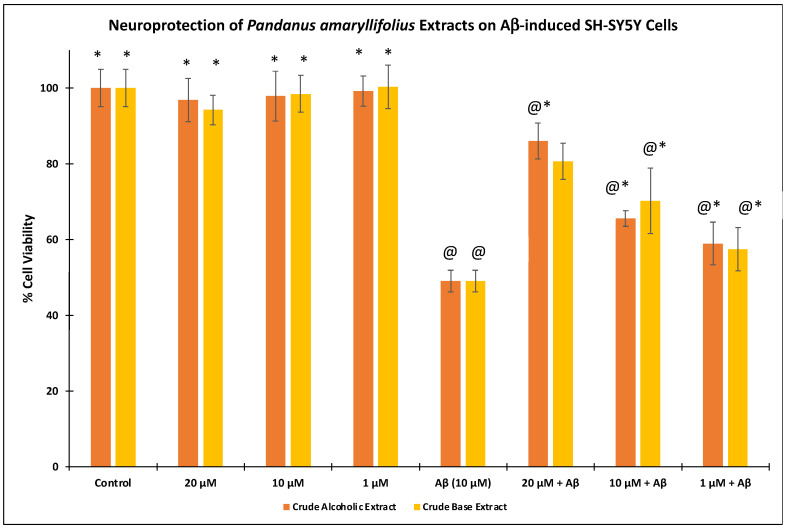
Effect of *Pandanus amaryllifolius* extracts on neuroprotective activity assay. Neuroblastoma SH-SY5Y cells were treated with the extracts for 6 h, followed by Aβ (10 μM) for 24 h. The % cell viability represents mean ± SD (triplicate experiments) and computed based on the control (untreated, 100%). @—statistically significant (*p* < 0.05) vs. control; *—statistically significant (*p* < 0.05) vs. Aβ-treated cells.

In the Aβ-insulted SH-SY5Y cytotoxicity assay (Figure 3), 49.05 ± 2.86% cell viability was observed following the treatment of 10 μM Aβ. Pre-treatment of the extracts at 1–20 μg/mL followed by 10 μM Aβ significantly (*p* < 0.05) restored the cell viability. Both extracts at 20 μg/mL showed the highest % cell viabilities at 86.02 ± 4.76% (CAE) and 80.65 ± 4.81% (CBE).

### 3.3. Level of Intracellular Reactive Oxygen Species (ROS)

**Figure 4:**
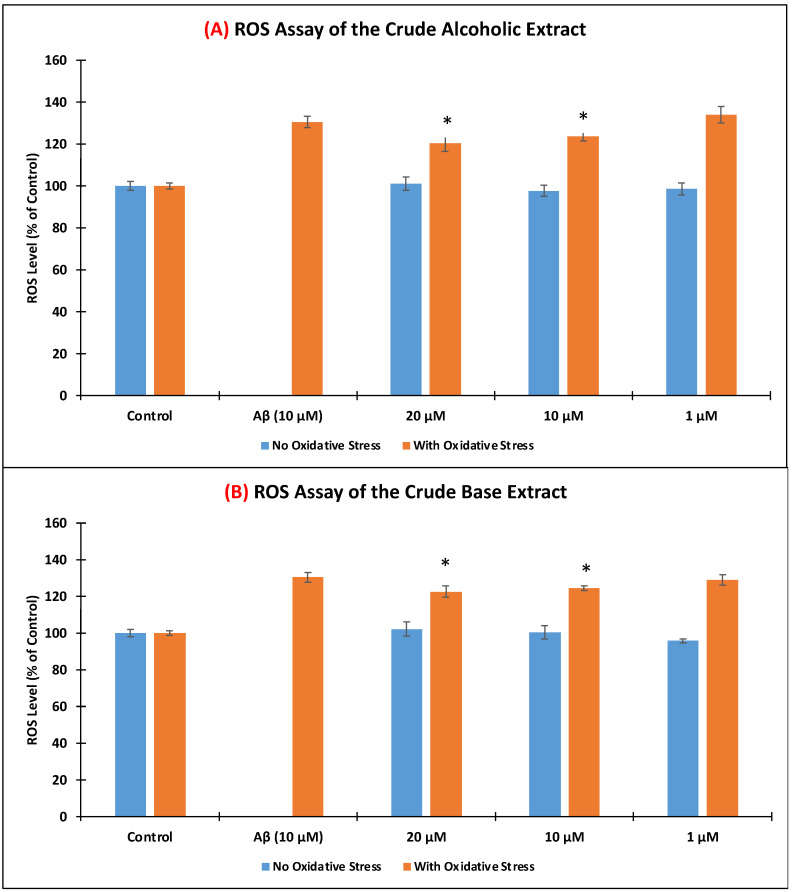
Effects of *Pandanus amaryllifolius* (**A**) crude alcoholic extract (**A**,**B**) crude base extract on intracellular ROS accumulation using the 2′,7′-dichlorodihydrofluorescein diacetate (H_2_DCFDA) reagent. Neuroblastoma SH-SY5Y cells were incubated with the extracts for 6 h, followed by 10 μM Aβ for 24 h. “No Oxidative Stress” indicates non-Aβ treated cells. Intracellular ROS level (% of the control cells) was computed as mean ± SD of triplicate experiments. The (*) represents statistical difference (*p* < 0.05) of the ROS level with the Aβ-treated alone cells.

The amount of intracellular ROS production when SH-SY5Y cells were induced with 10 μM Aβ was determined using 2′,7′-dichlorodihydrofluorescein diacetate (H_2_DCFDA) reagent. As shown in Figure 4, the level of ROS was comparable with the control when the cells were treated with only the crude extracts (blue bars). Upon incubation only with 10 μM Aβ, 130.52 ± 2.63% ROS level was generated. Upon pre-treating the SH-SY5Y cells with the crude extracts for 6 h at 10 and 20 μg/mL, a significant reduction (*p* < 0.05) in the ROS level was observed when compared to the SH-SY5Y cells treated only with Aβ. CAE exhibited 120.31 ± 3.77% (20 μg/mL) and 123.56 ± 2.04% (10 μg/mL) reductions, while CBE showed 122.63 ± 3.06% (20 μg/mL) and 124.52 ± 1.05% (10 μg/mL) ROS levels. Both extracts did not efficiently lower the ROS level at 1 μg/mL.

### 3.4. Effect of the P. amaryllifolius Extracts on the Mitochondrial Membrane Potential (Δψm)

**Figure 5:**
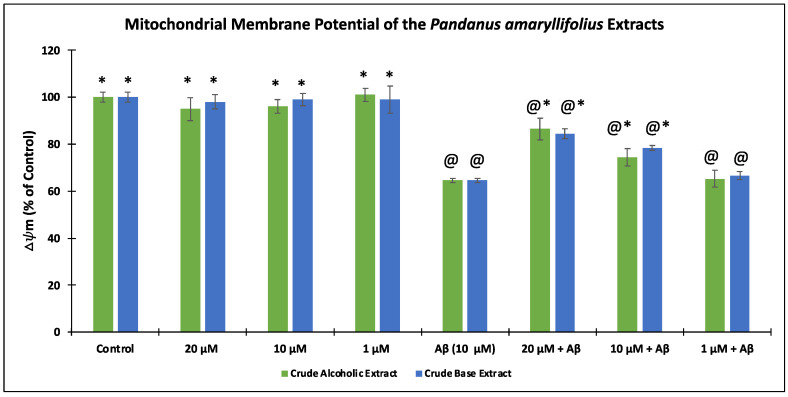
Assessment of the mitochondrial membrane potential (∆Ψm) of *Pandanus amaryllifolius* extracts using the tetramethylrhodamine methyl ester (TMRE) assay. SH-SY5Y cells were pre-treated with the extracts for 6 h, followed by Aβ (10 μM) for 24 h. The ∆Ψm (% of the control cells) was expressed as the mean ± SD of triplicate measurements. @—statistically significant (*p* < 0.05) vs. control; *—statistically significant (*p* < 0.05) vs. Aβ-treated cells.

As a result of Aβ induction in the neuroblastoma cells, an increase in ROS level was observed, which may result in mitochondrial dysfunctions. Hence, the capability of *P. amaryllifolius* extracts to restore the MMP was disclosed in Figure 5. Upon treatment with 10 Aβ only, SH-SY5Y cells gave 64.62 ± 3.21% Δψm. Pre-treatment with the extracts for 6 h prior to incubation with Aβ significantly (*p* < 0.05) restored the Δψm at 20 μg/mL (86.54 ± 4.61% for CAE and 84.55 ± 2.08% for CBE) and 10 μg/mL (74.43 ± 3.77% for CAE and 78.31 ± 1.06% for CBE). None of the extracts gave a substantial Δψm result at 1 μg/mL.

### 3.5. Isolation and Identification of Nicotinamide from the Crude Base Extract

Fractionation of *P. amaryllifolius* CBE led to the isolation of white crystals elucidated as nicotinamide by spectroscopic analyses. This is the first report on the isolation of nicotinamide, a pyridine-containing alkaloid, from a *Pandanus* species. Nicotinamide was previously identified to influence a flower-inducing activity in *Lemna gibba* G3 [28] and was elaborated to be a bioactive repellent against the blue mussel *Mytilus edulis* in *Mallotus japonicus* [29]. Nicotinamide was also identified in functional food plants such as dried peas, red lentils, and chickpeas by the HPLC method using a standard sample [30].

Although nicotinamide was not subjected to bioassay experiments in this study, foregoing in vitro and in vivo studies, have described its potential effects against AD. Nicotinamide restored cognitive deficits in vivo using the 3x-Tg AD mice model by selectively reducing Thr231 in a Tau protein associated with microtubule depolymerization and increasing the acetylated α-tubulin [31]. In vitro cell models showed that nicotinamide prevented mitochondrial and autophagy dysfunctions and reduced neuronal susceptibility to Aβ toxicity and oxidative and metabolic inducers [32]. In vivo results also indicated that 3xTgAD mice treated with nicotinamide for 8 months showed an improved cognitive function and a reduction in the Aβ and hyperphosphorylated tau proteins in the hippocampus and cerebral cortex [32]. Nicotinamide-treated transgenic 5xFAD mice resulted in the attenuation of deficits in spine density derived from primary hippocampal neurons of the mice models [33]. Furthermore, nicotinamide is subjected (12 July 2017–30 August 2022) to a Phase 2 clinical trial to test its effects in adults with mild cognitive impairment or mild Alzheimer’s disease [34,35].

## 4. Discussion

As part of our interest in exploring plant resources with anti-amyloidogenic activity against AD, we have investigated the crude alcoholic and crude alkaloidal extracts of *P. amaryllifolius*. *P. amaryllifolius* is an important medicinal plant in Philippine traditional folk medicine. Its ethnomedicinal uses were scientifically validated in pharmacological studies including anti-inflammatory [36], antioxidant [17], antiproliferative [17], hepatoprotective [37], antidiabetic [38], antimicrobial [39], and antiviral [40] properties. The anti-cholinesterase and β-secretase activities of a commercially available tea infusion of *P. amaryllifolius* (Pandan herbal tea) from Thailand have been reported [41]. Thus far, the anti-amyloidogenic and neuroprotective effects on Aβ toxicity of *P. amaryllifolius* are still unknown. Recently, we described the neuroprotective activity of *P. clementis* [22] and the identification of phytosterols as possible bioactive agents [42]. *P. amaryllifolius* is also an excellent source of alkaloids, collectively known as *Pandanus* alkaloids, yet biological studies on these *Pandanus* alkaloids, including the crude alkaloids extract, are limited [16,39]. Hence, this study also contributes to the limited pharmacological information specifically focused on the alkaloidal fraction of *P. amaryllifolius*. Natural products, particularly alkaloids, have been the primary source of new scaffolds for the treatment of neurodegenerative diseases, including AD. Interestingly, the current drugs memantine, tacrine, rivastigmine, donepezil, and galantamine approved by the US FDA for AD are all alkaloids.

In the present work, we were able to disclose the alkaloidal extract of *P. amaryllifolius* to effectively inhibit the aggregation and oligomerization of Aβ. As a major contributor leading to AD, these Aβ aggregates lead to Aβ plaques causing various oxidative stressors, neuroinflammation, mitochondrial deterioration, and death. Hence, the neuroprotective effects of *P. amaryllifolius* extracts on Aβ-insulted neuronal cells were also assessed. As seen in Figure 3, *P. amaryllifolius* extracts rescued the cell damage caused by Aβ toxicity. This may be explained by reducing the levels of ROS and increasing the mitochondrial membrane potential. To further explore the possible bioactive scaffold, the alkaloid fraction was subjected to chromatographic purification. Unprecedentedly, we were able to isolate nicotinamide, a pyridine-type compound that possesses an array of in vitro and in vivo activities against AD [31,32,33]. Our results also corroborated the previous report [41] on *P. amaryllifolius* as a potential medicinal plant against AD. Since nicotinamide was isolated as a minor metabolite comprising only 0.06% of the crude alkaloid fraction, we may speculate that the *Pandanus* alkaloids may have synergistic inhibitory and neuroprotective effects and other potential bioactive *Pandanus* alkaloids are present in the extract. Hence, comprehensive isolation of known and possibly new *Pandanus* alkaloids is being undertaken to assess their in vitro activities against Aβ aggregation and toxicity. Moreover, inhibition of Aβ aggregation and oligomerization was evaluated using protein-based assays. From a future perspective, Aβ will transfect neuronal cells to exploit the anti-aggregation and anti-oligomerization of plant extracts and pure compounds in live cells. Furthermore, validation of the neuroprotective effects of the alkaloids on other neuronal cells and exploring other AD-related mechanistic assays are also warranted.

## Figures and Tables

**Table 1:** Measurement of the Inhibition of Amyloid-β Aggregation using the Thioflavin T (ThT) Assay and the Inhibition of Amyloid-β Oligomerization using the Multimer Detection System (MDS) Assay.

	ThT Assay ^a^ (% Inhibition) ^c^	MDS Assay ^b^ (Optical Density) ^c^
Crude Alcoholic Extract	73.67 ± 3.54	2.559 ± 0.19 ^
Crude Base Extract	89.53 ± 5.21 *	2.027 ± 0.080 ^
Phenol Red (Positive Control)	70.06 ± 2.87	
BPL-1 (Positive Control)		1.509 ± 0.18 ^
Negative Control		2.203 ± 0.068

^a^ The extracts and positive control were measured at 50 μg/mL and 50 μM, respectively. ^b^ The extracts and positive control were measured at 100 μg/mL and 100 μM, respectively. ^c^ The % inhibition and optical density are expressed as mean ± SD of triplicate experiments. * Significant difference at *p* < 0.05 versus the positive control (phenol red). ^ Significant difference at *p* < 0.05 versus the negative control.

## Data Availability

Not applicable.

## Supplementary Materials

The following supporting information can be downloaded at: https://www.mdpi.com/article/10.3390/nu14193962/s1, NMR and MS spectra of nicotinamide.
