# Supplementary material for: Pandanus amaryllifolius Exhibits In Vitro Anti-Amyloidogenic Activity and Promotes Neuroprotective Effects in Amyloid-β-Induced SH-SY5Y Cells
Source: Nutrients. 2022 Sep 24;14(19):3962. doi: 10.3390/nu14193962 (PMC9571295; doi:10.3390/nu14193962)
Supplement: Supplementary file 1 [file nutrients-14-03962-s001.zip › nutrients-1888541-supplementary.pdf]

Supplementary Material

Pandanus amaryllifolius Exhibits *In vitro* Anti-Amyloidogenic Activity and Promotes Neuroprotective Effects in Amyloid-β-Induced SH-SY5Y Cells

Mario A. Tan[1*], Hayato Ishikawa[2], Seong Soo A. An[3*]

[1]College of Science and Research Center for the Natural and Applied Sciences, University of Santo Tomas, España, Manila 1015 Philippines

[2]Graduate School of Pharmaceutical Sciences, Chiba University, 1-8-1 Inohana, Chuo-ku, Chiba 260-8675 Japan

[3]Bionano Research Institute, Department of Bionano Technology, Gachon University, Seongnam-si, Gyeonggi-do, Republic of Korea

*Correspondence: matan@ust.edu.ph (MAT); seongaan@gachon.ac.kr (SSAA)

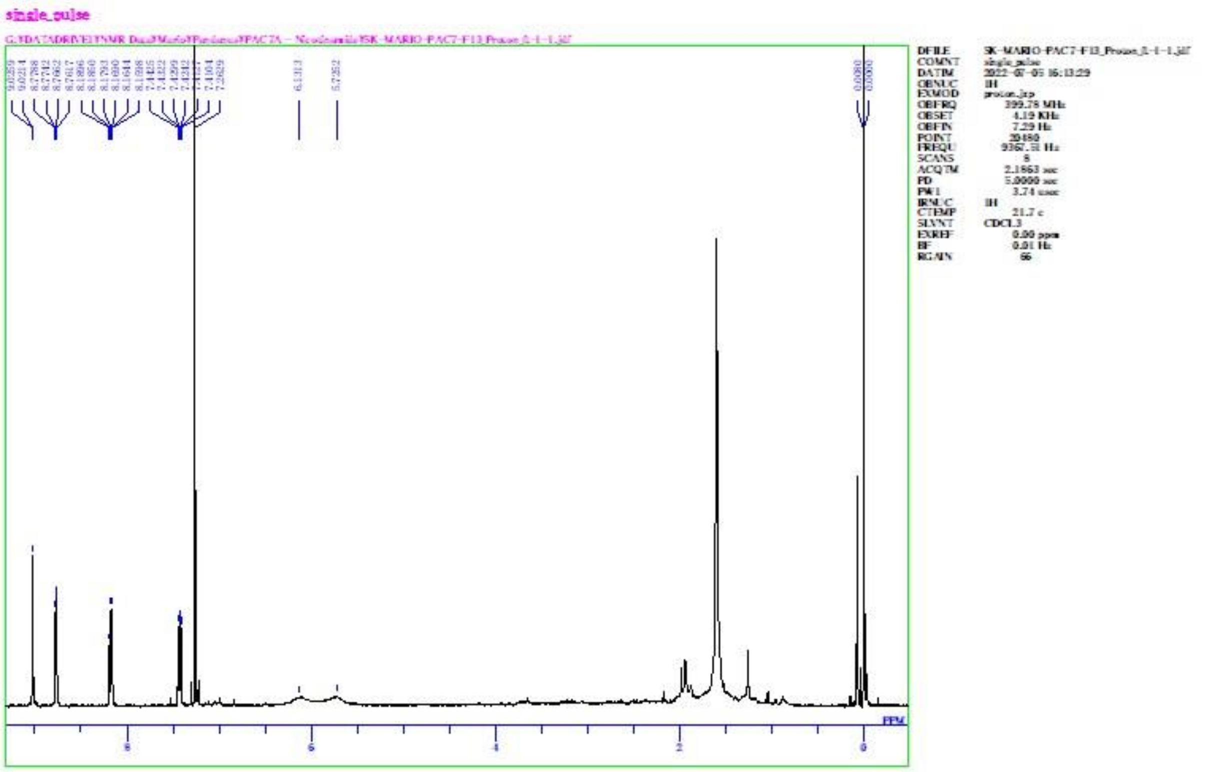

^{1}H NMR of Nicotinamide (400 MHz, $CDCl_3$)

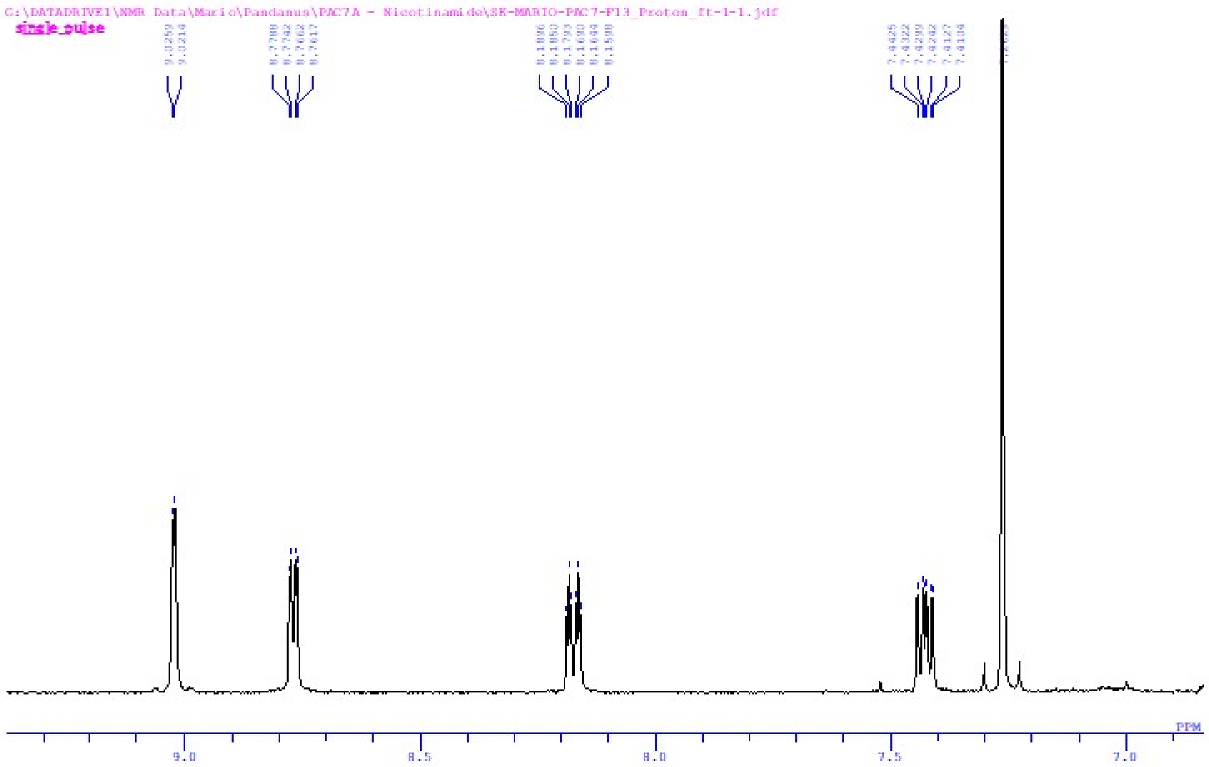

Expansion of the ^{1}H NMR of Nicotinamide (400 MHz, $CDCl_3$)

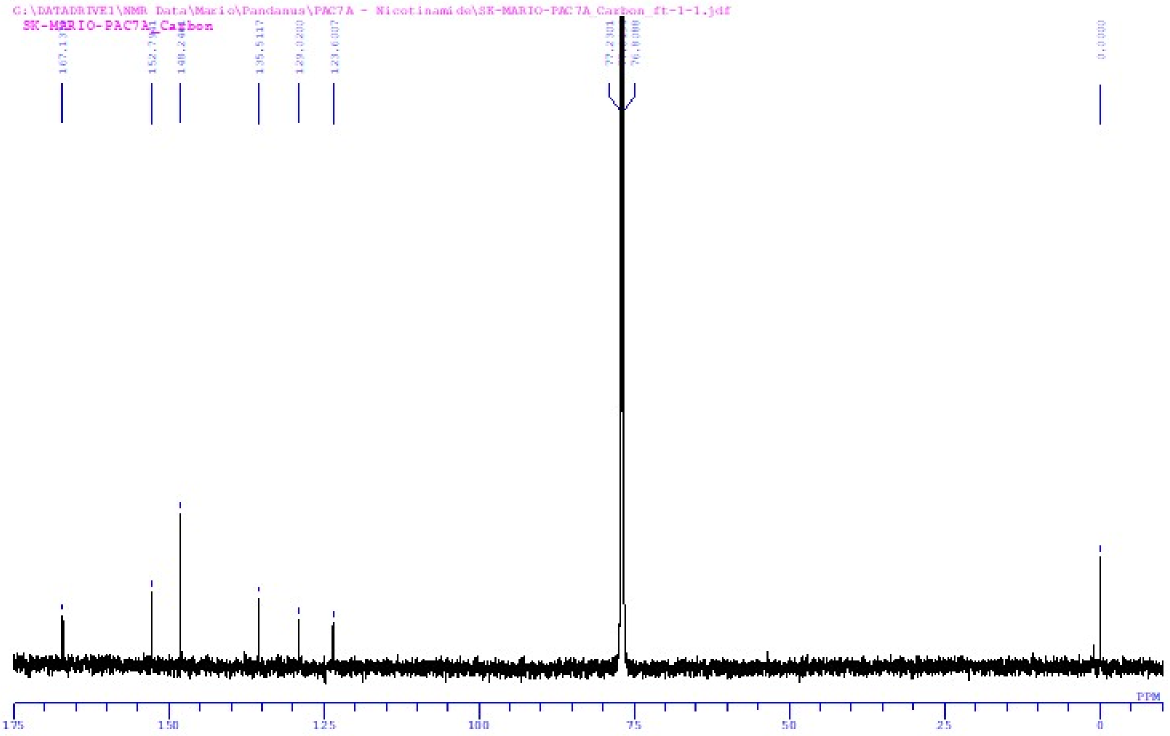

^{13}C NMR of Nicotinamide (150 MHz, $CDCl_3$)

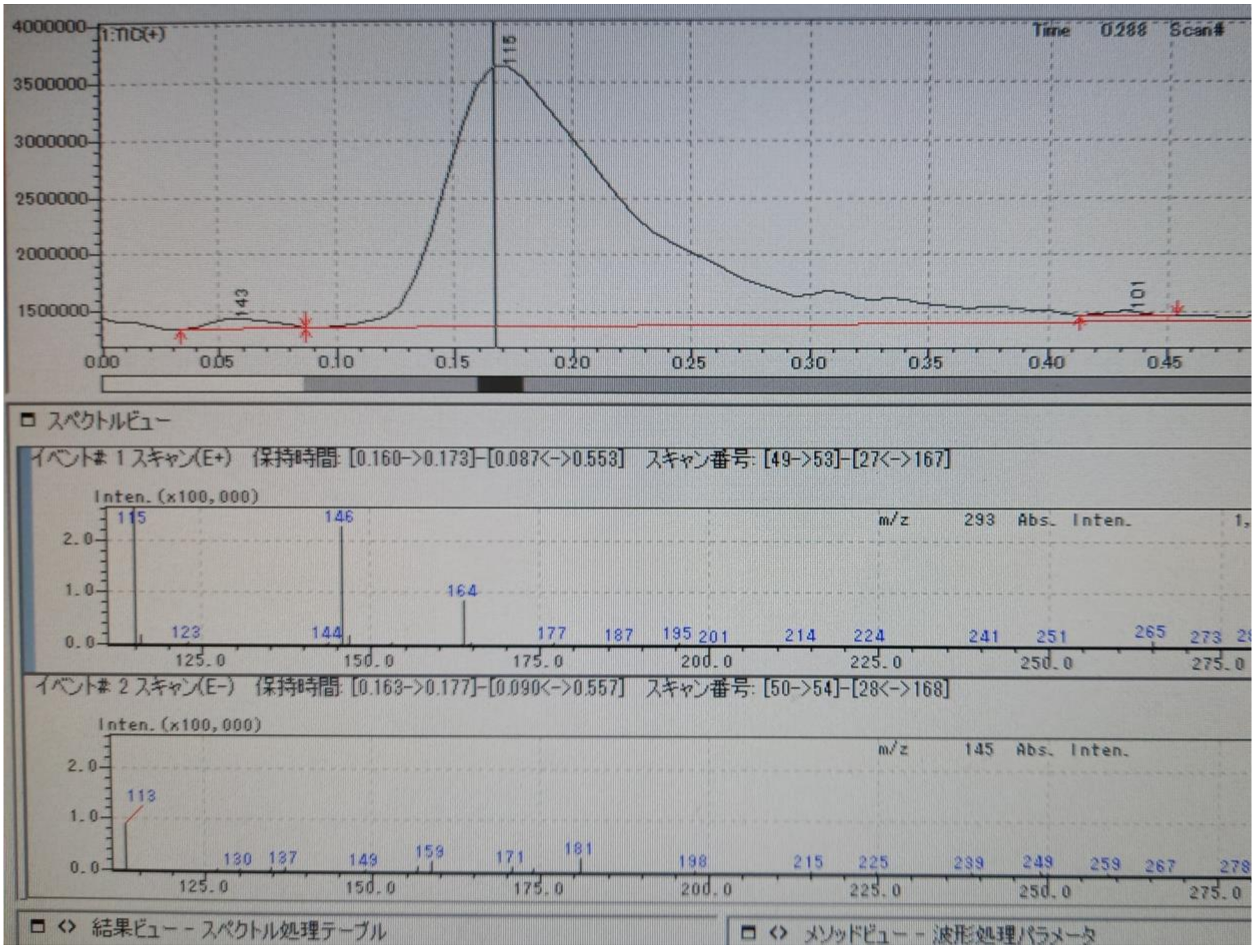

LC-MS spectra of Nicotinamide

Liquid Chromatograph Mass Spectrometer
LCMS-2020 (Shimadzu)
